# Investigation on the incidence and risk factors of lung cancer among Chinese hospital employees

**DOI:** 10.1111/1759-7714.14549

**Published:** 2022-07-11

**Authors:** Zi‐Hao Chen, Zhi‐Yong Chen, Jing Kang, Xiang‐Peng Chu, Rui Fu, Jia‐Tao Zhang, Yi‐Fan Qi, Jing‐Hua Chen, Jun‐Tao Lin, Ben‐Yuan Jiang, Xue‐Ning Yang, Yi‐Long Wu, Wen‐Zhao Zhong, Qiang Nie

**Affiliations:** ^1^ School of Medicine South China University of Technology Guangzhou China; ^2^ Guangdong Lung Cancer Institute, Guangdong Provincial People's Hospital Guangdong Academy of Medical Sciences Guangzhou China; ^3^ The Second School of Clinical Medicine Southern Medical University Guangzhou China; ^4^ 12th People's Hospital of Guangzhou Guangzhou China

**Keywords:** early screening, ionizing radiation, PM2.5, pulmonary nodules, risk factors

## Abstract

**Objective:**

In recent years, the lung cancer incidence has grown and the population is younger. We intend to find out the true detection rate of pulmonary nodules and the incidence of lung cancer in the population and search for the risk factors.

**Method:**

Hospital employees ≥40 years old who underwent low‐dose computed tomography (CT) lung cancer screening from January 2019 to March 2022 were selected to record CT‐imaging characteristics, pathology, staging, and questionnaires to investigate past history, smoking history, diet, mental health, etc. PM2.5 and radiation intake in radiation‐related occupation received monitoring in hospital.

**Result:**

The detection rate of suspicious pulmonary nodules was 9.1% (233/2552), and the incidence rate of lung cancer (including adenocarcinoma in situ) was 4.0% (103/2552). Morbidity among doctors, nurses, technicians, administers, and logistics was no difference (*p* = 0.184), but higher in women than in men (4.7% vs 2.4% *p* = 0.002). The invasiveness increased with age and CT density of nodules (*p* = 0.018). The relationship between lung cancer morbidity and PM2.5 was not clear (*p* = 0.543); and no lung cancer has been found in employees related ionizing radiation.

**Conclusion:**

The high screening rate has brought about a high incidence of lung cancer. At present, the risk factor analysis of lung cancer based on small samples cannot find the direct cause. Most of the ground glass opacity (GGO)s detected by LDCT screening are indolent, but there are also rapidly progressive lung cancer. A predictive model to identify active and indolent GGO is necessary.

## Graphical abstract

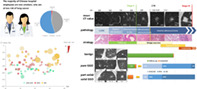

## INTRODUCTION

According to relevant data released, the number of lung cancer cases in China has exceeded 830 000 in 2016, and among all related cases, the death rate has reached 660 000.^1^ The incidence of lung cancer ranks first among cancers.^2^ The prevention and treatment of lung cancer is a major challenge in China.^3^, ^4^, ^5^, ^6^, ^7^ Therefore, early detection of lung cancer through screening can effectively improve the prognosis of lung cancer and significantly reduce the mortality rate of lung cancer.^8^, ^9^, ^10^, ^11^

The current evidence that low‐dose helical computed tomography (LDCT) reduces lung cancer mortality comes primarily from two randomized controlled trials.^8^, ^12^, ^13^, ^14^ From 2002 to 2004, the National Lung Screening Trial (NLST) was carried out by the American Lung Cancer Screening Research Group.^10^, ^15^, ^16^, ^17^ A total of 53 454 people at high risk of lung cancer were included, and they were randomly divided into LDCT group and chest X‐ray group. Compared with chest X‐ray, LDCT can reduce lung cancer mortality by 20%. During this period, Helen^18^, ^19^, ^20^, ^21^ first carried out a lung cancer screening test on patients. 13 195 men and 2594 women were included. The subjects were all at high risk of lung cancer and were randomly divided into four rounds of LDCT screening or no screening. The results showed that the 10‐year cumulative mortality from lung cancer was reduced by 24% for men in the screening group and 33% for women.

According to previous research reports, it is known that repeated LDCT screening in high‐risk groups of lung cancer can effectively reduce the death of patients because of lung cancer to a certain extent. However, considering the epidemiological characteristics of lung cancer in different regions and the differences in the availability of medical resources,^19^, ^22^, ^23^, ^24^, ^25^, ^26^ whether high‐risk groups with different definitions can also benefit from one‐time LDCT screening is an urgent scientific question that needs to be answered.^27^, ^28^, ^29^, ^30^, ^31^

### The effect of popularization of LDCT on the detection rate of lung cancer

Chest X‐rays are usually performed in routine physical examinations. This examination has great limitations in the detection of pulmonary nodules, and it is easy to miss the sub‐centimeter lesions of pulmonary nodules, approximately 22% to 85% of early lung cancers may be miss detection. For lung cancer, LDCT screening is more sensitive and can more effectively detect smaller, earlier lung cancers than chest X‐rays. The NLST study group conducted LDCT screening, and the control option was chest X‐ray. LDCT once a year for a total of 3 years can reduce the probability of lung cancer death by 0.33% and the relative risk by 20%. The most promising lung cancer screening technology today is LDCT. In terms of the ability to detect small nodules in the lungs, it is 10 times higher than that of chest X‐rays, and it can detect small lung cancers with a diameter of <1 cm and only local infiltration without distant metastasis, 80% to 90% of tumors can be cured with adequate surgical resection. The advantages of LDCT are obvious, including: (1) early detection—it can detect small lesions of early lung cancer, and at the same time can detect other chest tumors, some vascular lesions radiation, and non‐tumor lesions; (2) low dose—uses only ~10% of the conventional CT radiation dose; (3) fast and non‐invasive—scanning is completed within 10 seconds and is non‐invasive; (4) high detecting probability and diagnostic accuracy—it can show more than 90% match with the postoperative pathology.^32^, ^33^, ^34^, ^35^, ^36^, ^37^, ^38^

### The relationship between invasiveness and CT features

At present, most of the malignant pulmonary ground glass nodules are lung adenocarcinoma, and lung adenocarcinoma is divided into lung atypical adenomatous hyperplasia (AAH), adenocarcinoma in situ (AIS), minimally invasive lung adenocarcinoma (MIA), and invasive lung adenocarcinoma (IAC). There is still controversy as to which stage of lung adenocarcinoma requires surgery, but once the ground glass nodule is highly suspected to be IAC, surgery is definitely suitable, because IAC is atypical compared to lung adenomatous hyperplasia, carcinoma in situ, and MIA. The prognosis will be significantly worse, and the risk of metastasis will also increase, so the accurate prediction of IAC is the most clinically meaningful. For sub‐centimeter ground glass nodules, CT value measurement is convenient and can quantify the density of expression lesions. It can not only estimate the subtype of lung adenocarcinoma, but also quantify the progress of expression lesions during follow‐up. In clinical work, it is widely used. According to the lesion density measurement, we can accurately subtype diagnosis of lung adenocarcinoma.

### Additional risk factors

Occupational exposures of different origin probably comprise the third cause of lung cancer around the world. Additional risk factors, which have been well‐studied, include exposure to radon,^39^ occupational hazards, domestic fuel smoke (biomass), and infectious diseases. The following section overviews each factor and its association to lung carcinogenesis.^36^, ^40^, ^41^, ^42^

Smoking prevalence has been lower in women in comparison to men in many countries, yet this difference has been narrowing down in many parts of the world. When analyzing lung cancer in never smoker patients, the diagnosis is most frequently found in females, but mortality rates because of this disease tend to be very similar between them. Female patients are also more frequently diagnosed with the most common driver mutation abnormalities such as Epidermal growth factor receptor (EGFR) mutation, EML4‐ALK fusion, and ROS‐1 mutation, which have also been associated with a low smoking history of patients. If there is a gender difference in the pathogenesis of lung cancer because of genetic aspects of women, or if this is a matter of differences in environmental exposures compared to men, remains a valid, but difficult question to answer.

## METHODS

A total of 2107 employees ≥40 years old that underwent LDCT lung cancer screening in Guangdong Provincial People's Hospital from January 2019 to March 2022 were enrolled in the study. LDCT is tested voluntarily and employees who find pulmonary nodules made their own decision about surgical resection. The threshold size of positive pulmonary nodules detected by LDCT was ≥3 mm. Lung nodules detected by LDCT were managed according to the National Comprehensive Cancer Network (NCCN) guidelines. The medical examination center recorded the employees who are found to be positive for small nodules on LDCT and sent the list to the Department of Pulmonary Oncology to decide whether to proceed with the next intervention. In fact, most employees with suspected malignant nodules are still being followed up. During the screening, the age, gender, smoking status, previous detection of pulmonary nodules, and family history of tumor history of the employees participating in the screening will also be simply recorded.

### Decision‐making for pulmonary nodules

**FIGURE 1:**
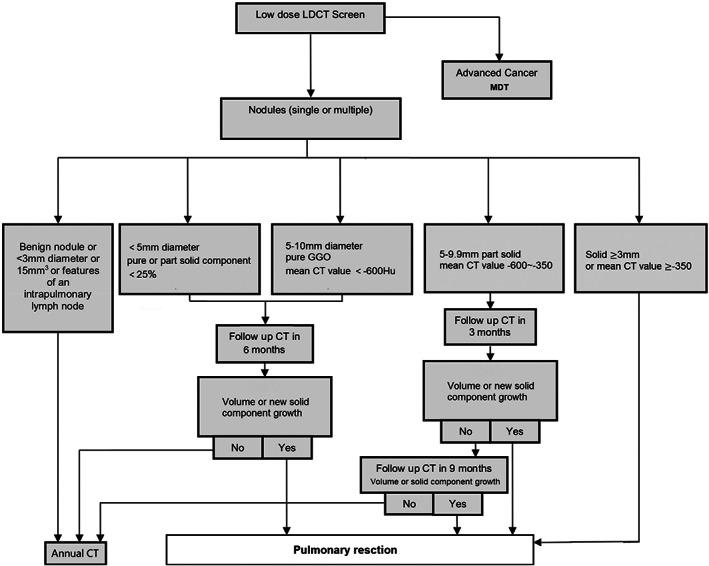
Decision‐making for pulmonary nodules.

For nodules <3 mm diameter, it is recommended to review CT after 1 year, for nodules <5 mm, solid components <25%, or 5 to 10 mm diameter pure ground glass opacity (GGO) (with mean CT value < −600 Hu) nodules, it is recommended to review CT after 6 months. For 5 to 10 mm part‐solid nodules, it is recommended to review CT after 3 months. For solid nodules ≥8 mm or solid components ≥5 mm in part‐solid nodules, enhanced chest CT or positron emission tomography (PET)‐CT is recommended. If lung cancer is highly suspected, biopsy or surgery should be performed to confirm the pathology. Regarding the follow‐up time limit, existing research data show that if there is no progress in ground glass nodules with mixed density after 3 years of follow‐up, there is no need to continue follow‐up; pure ground glass nodules require longer follow‐up to observe their possible progression. The primary objective of repeat screening was to identify all noncalcified nodules describe interval growth. Growth was defined as an interval increase in the diameter of the nodules in any direction as determined on the cross‐sectional CT images. Annual repeat scans were regarded as positive if a pre‐existing nodule was found to have grown or if a new nodule was detected. If growth was observed after the follow up period(s), options included resection (Figure 1).

### Questionnaire design and collection

Employees in our hospital who were over 40 years old (including 40 years old) starting chest LDCT screening in 2019. The patients were divided into a suspected malignant nodules group and benign group according to the CT image features. The suspected malignant nodules group was divided into a postoperative pathology confirmed group and following up group. Epidemiological questionnaires were collected by an experienced surgeon during the follow‐up, including age, gender, race, marital status, smoking status, alcohol consumption, dietary habits, occupational exposure, family history of cancer, nonpulmonary chronic diseases, pre‐existing lung disease, dwelling environment exposure, and so on. Smoking status was divided into four categories: no smoking, passive smoking, mild smoking, and heavy smoking. According to the definition of the World Health Organization (WHO), passive smoking meant that non‐smokers had inhaled the smoke exhaled by smokers for at least 15 minutes more than 1 day in a week. The degree of smoking in current and former smokers was measured by heaviness of smoking index (HSI),^11^ which was <400 for mild smoking, and ≥400 for heavy smoking. Alcohol consumption was assessed using the consumption subscale of the Alcohol Use Disorders Identification Test (AUDIT). Dietary habits were divided into healthy diet or unhealthy diet. A diet of moderation rich in fruits and vegetables was defined as the healthy diet. Family history of cancer was divided into three categories: no, other cancer family history, and lung cancer family history. Preexisting lung diseases included pneumonia, emphysema, asthma, chronic bronchitis, pulmonary fibrosis, tuberculosis, and chronic obstructive pulmonary disease.

### Quantitative monitoring of PM2.5 and ionizing radiation

Every 10 000 m^2^ office area is equipped with a dust measuring instrument (25 in total, 24 hour a day continuous uninterrupted monitoring) to collect data from July 2021 to March 2022. Each dust measuring instrument is linked by the wireless 5G network in the hospital and independently records the dust concentration in the jurisdiction and the value is represented by a grayscale image. Every 1 minute the cenral host send the latest data, and it is recorded on the hard disk to produce PM2.5 curve according to the high points on dust concentration contour map, according to the density of the contour map, the regional dust concentration heat map is made. The dynamic and uninterrupted recording can accurately reflect the dust concentration difference and proportion change between regions.

Thirty hospital employees engaged in ionizing radiation were asked to long‐term carry a personal radioactivity measurement material (Thermo Scientific TLD) for dynamic monitoring of radiation exposure during work, and it would sound an alarm when the radiation exceeds a safe value. The total annual dose intake is calculated by multiplying the daily working hours intake by the number of working days per year.

## RESULTS

**FIGURE 2:**
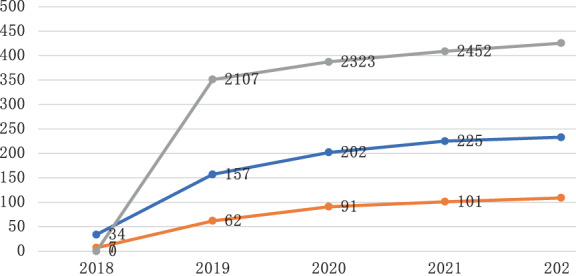
The annual cumulative number of physical examinations, the number of suspicious pulmonary nodules detected, and the number of resections are line graphs.

In 2019, a total of 2107 employees participated in the screening, accounting for 73.8% (2107/2855) of the total number of employees over the age of 40. Among them, 157 cases of suspicious lung nodules were found, accounting for 7.5% (157/2107) of the total number of people screened, and 2.9% (62/2107) cases underwent surgical resection of lung nodules. In 2020, a total of 2323 employees participated in the screening, accounting for 81.4% (2323/2855) of the total number of employees over the age of 40. Among them, 8.7% (202/2323) cases of suspicious pulmonary nodules were found, and 3.9% (91/2323) cases underwent surgical resection of pulmonary nodules. In 2021, a total of 2452 employees participated in the screening, accounting for 85.9% (2452/2855) of the total number of employees over the age of 40. Among them, 9.2% (225/2452) cases of suspicious pulmonary nodules were found, and 4.4% (109/2452) cases underwent surgical resection of pulmonary nodules. The employees who have participated in the screening will be tracked and registered. As of March 30, 2022, a total of 2552 employees have been registered, and 233 suspicious pulmonary nodules were found, including 139 pure ground glass nodules, 67 mixed density ground glass nodules, and 21 cases of solid nodules. A total of 124 cases were under following up and 109 cases were surgically removed, including six cases of benign nodules, 23 cases of AIS, 48 cases of MIA, 32 cases of IAC confirmed by pathology, and 103 cases of lung cancer in a broad sense. Estimated the incidence of lung cancer among employees over 40 years old was 103/2552 = 4.0%. The newly detected suspicious nodules were all from new members who had never participated in lung cancer screening before, and no new nodules appeared in the employees without suspicious nodules under following‐up (Table 1, Figure 2).

**TABLE 1:** The detection rate of suspicious nodules and the prevalence of lung cancer in employees of different age groups

Age	Lung cancer (%)	Detected (%)	Screened
40, 45	26 (2.92)	60 (6.75)	889
45, 50	35 (3.36)	90 (8.65)	1041
50, 55	31 (4.32)	61 (8.49)	718
55, 60	11 (4.20)	61 (8.39)	262

*Notes*: lung cancer: diagnosed by histology after surgery. Detected: suspicious pulmonary nodules found in LDCT.

The NCCN guidelines version 1.2022 recommends that individuals at high risk for lung cancer should be screened using LDCT, individuals at low risk are not recommended LDCT screening. The high risk and low risk individuals are defined as the following:

- High risk: individuals age 50 years or older with a 20 or more pack‐year history of smoking.
- Low risk: individuals younger than 50 years old and/or with a smoking history of fewer than 20 pack‐years.
- Moderate: not within the individuals of those recommended or not recommended LDCT by NCCN guidelines, including ages 50 years or older with a smoking history of fewer than 20 pack‐years, and ages younger than 50 years with a 20 or more pack‐year history of smoking.

**FIGURE 3:**
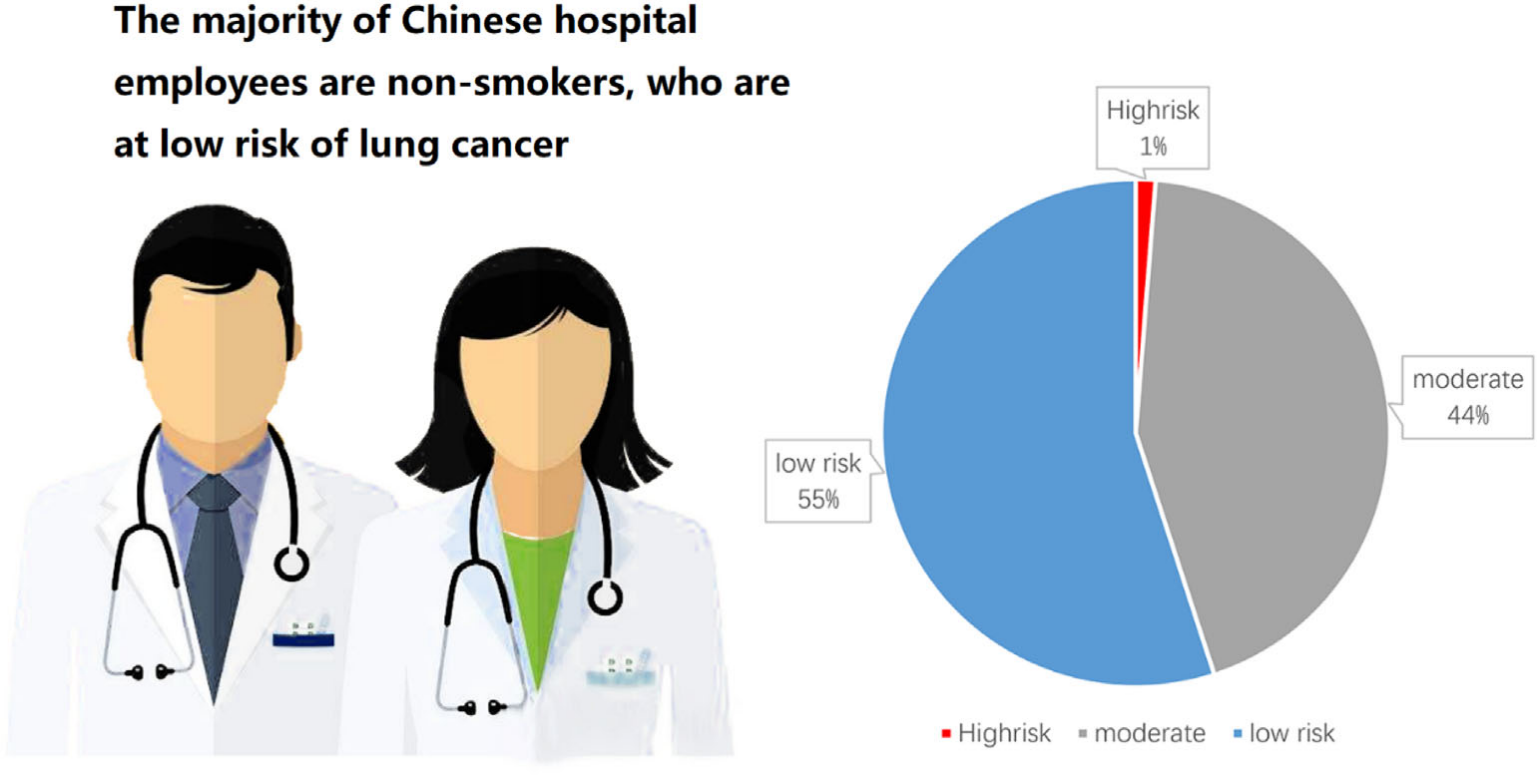
Risk for lung cancer among hospital employees.

In 103 patients with lung adenocarcinoma, the low risk and moderate were 57 and 45, respectively. Only one person belongs to the high risk individual according to NCCN recommends (Figure 3).

Among the 80 employees diagnosed with MIA and IAC, 78 were in stage IA, and 2 were in stage IIIA‐N2. Patients with stage IA were individually customized according to the location of the nodule and the specific degree of invasion. All patients underwent sublobar resection to protect lung function, and the scope of surgical resection was decided based on the experience of JCOG0802/0804. The patients achieved R0 resection and without adjuvant therapy after surgery; two patients with stage IIIA‐N2 were evaluated for surgery after neoadjuvant therapy, one patient was down staged and underwent R0 resection and finally benefited from neoadjuvant therapy, but the other patient progressed to IIIB‐N3 and considered unresectable, followed by concurrent chemoradiotherapy.

**FIGURE 4:**
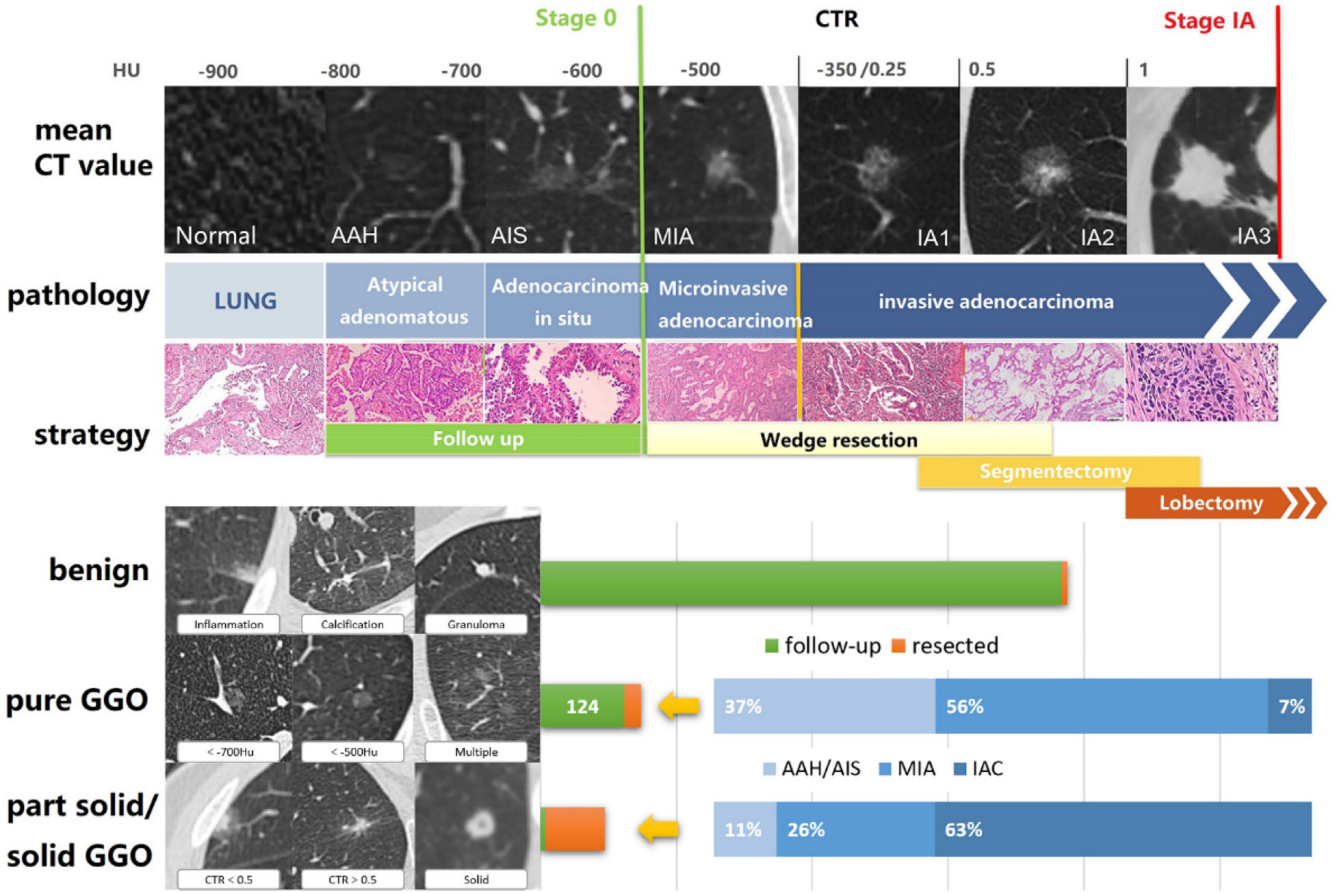
GGO types and proportions of nodules screened by LDCT, and the proportion of corresponding pathological types.

Among the 109 resected patients, classified according to the density of imaging CT values, 58 were pure ground‐glass nodules (pGGO), 39 were mixed‐density ground‐glass nodules (mGGO), and 15 of them were solid. (Figure 4).

**FIGURE 5:**
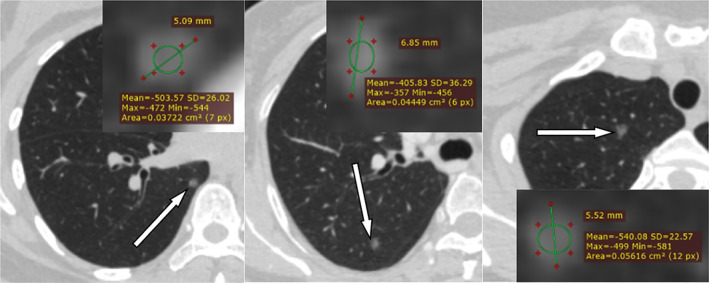
Imaging features of three invasive adenocarcinomas with pure ground glass nodules smaller than 6 mm.

**FIGURE 6:**
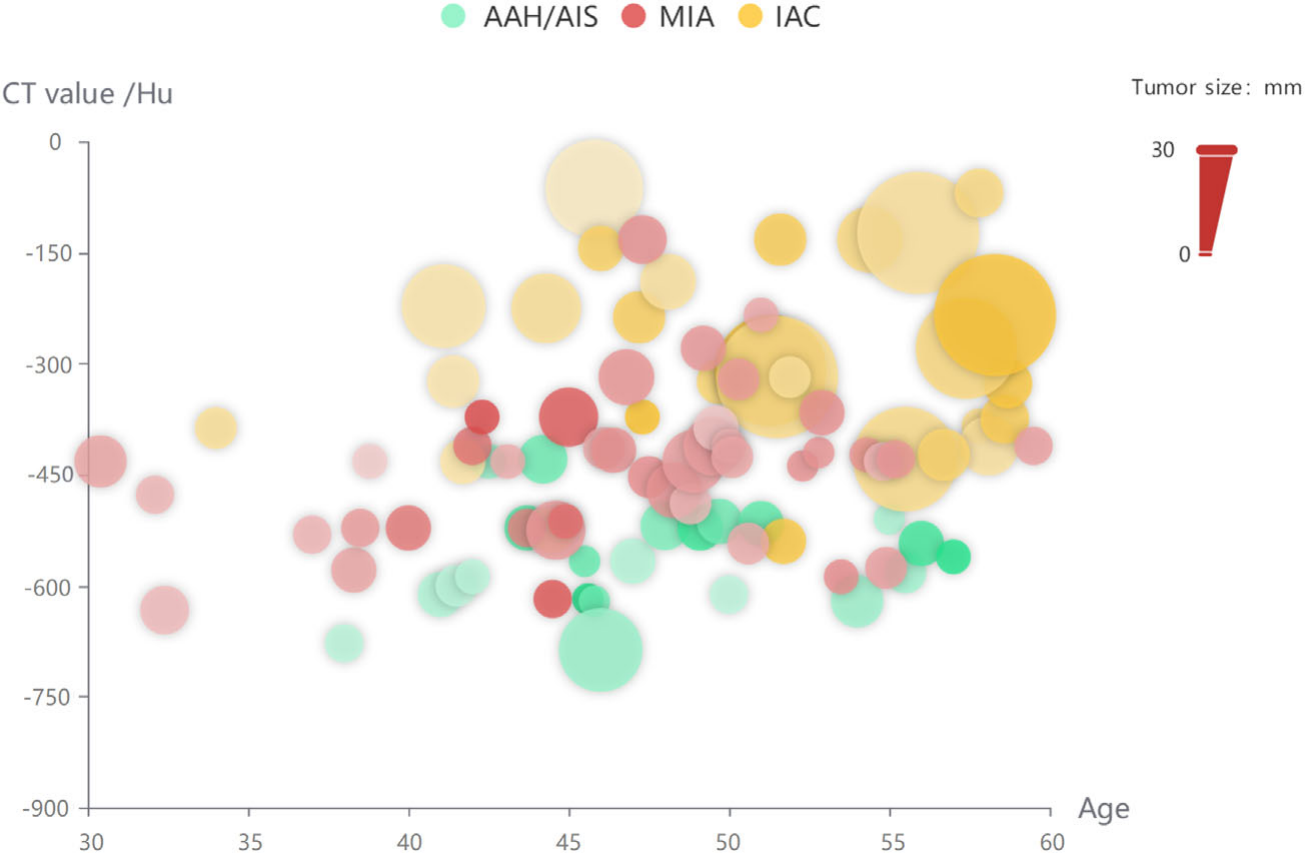
Imaging, pathology, age‐related bubble charts, the invasiveness of nodules increased with age and CT density of nodules (*p* = 0.018).

Three cases of pGGO pathology confirmed IAC, considered an early stage of rapidly progressive lung adenocarcinoma. (Figure 5) The invasiveness of nodules increased with age and CT density of nodules (Table 2, Figure 6).

**TABLE 2:** Comparison of ground glass composition ratio and wettability

	plGGO	phGGO	mGGO	Solid	<6 mm	6–10 mm	11–20 mm	21–30 mm	Total
Benign	0	2	0	3	0	5	0	0	5
AAH	1	0	0	0	0	0	1	0	1
AIS	14	5	4	0	7	16	0	0	23
MIA	8	24	15	1	15	28	5	0	48
IAC	0	4	18	10	3	7	20	2	32
total	23	35	37	14	25	56	26	2	109

Abbreviations: plGGO, pure low CT value ground glass nodules, mean CT value <−500 Hu; phGGO, pure high CT value ground glass nodules, mean CT value −500 to −350 Hu; Benign, inflammatory lesion; AAH, lung atypical adenomatous hyperplasia; AIS, adenocarcinoma in situ; MIA, minimally invasive lung adenocarcinoma; IAC, invasive lung adenocarcinoma.

### Comparison of clinical characteristics of pulmonary nodule surgery population and follow‐up population

Most pulmonary nodules in the employees with follow‐up strategies were pure ground glass nodules in 117/124 (94.0%), and 121/124 (97.6%) were nodules within 1 cm in diameter during follow‐up. There were 18 cases with increased density or increased volume, all of which underwent surgical resection. In the clinical treatment stage, the images of pulmonary nodules should be observed, and the corresponding cases should be selected for analysis. They can be divided into carcinoma in situ, microscopic invasive adenocarcinoma, IAC, and other related cases (Table 3).

**TABLE 3:** The resection and follow up state of suspicious nodule

Strategy	Resection immediately	Resection after review	Resection after progression	Under following
*n*	38	48	18	124
pGGO	19	34	5	117
mGGO	11	10	10	7
Solid	8	4	3	
<6 mm	8	14	1	38
6–10 mm	13	15	3	83
11–20 mm	17	17	14	3
21–30 mm		2		

### Occupation and gender distribution of lung cancer diagnosed population among hospital workers

**FIGURE 7:**
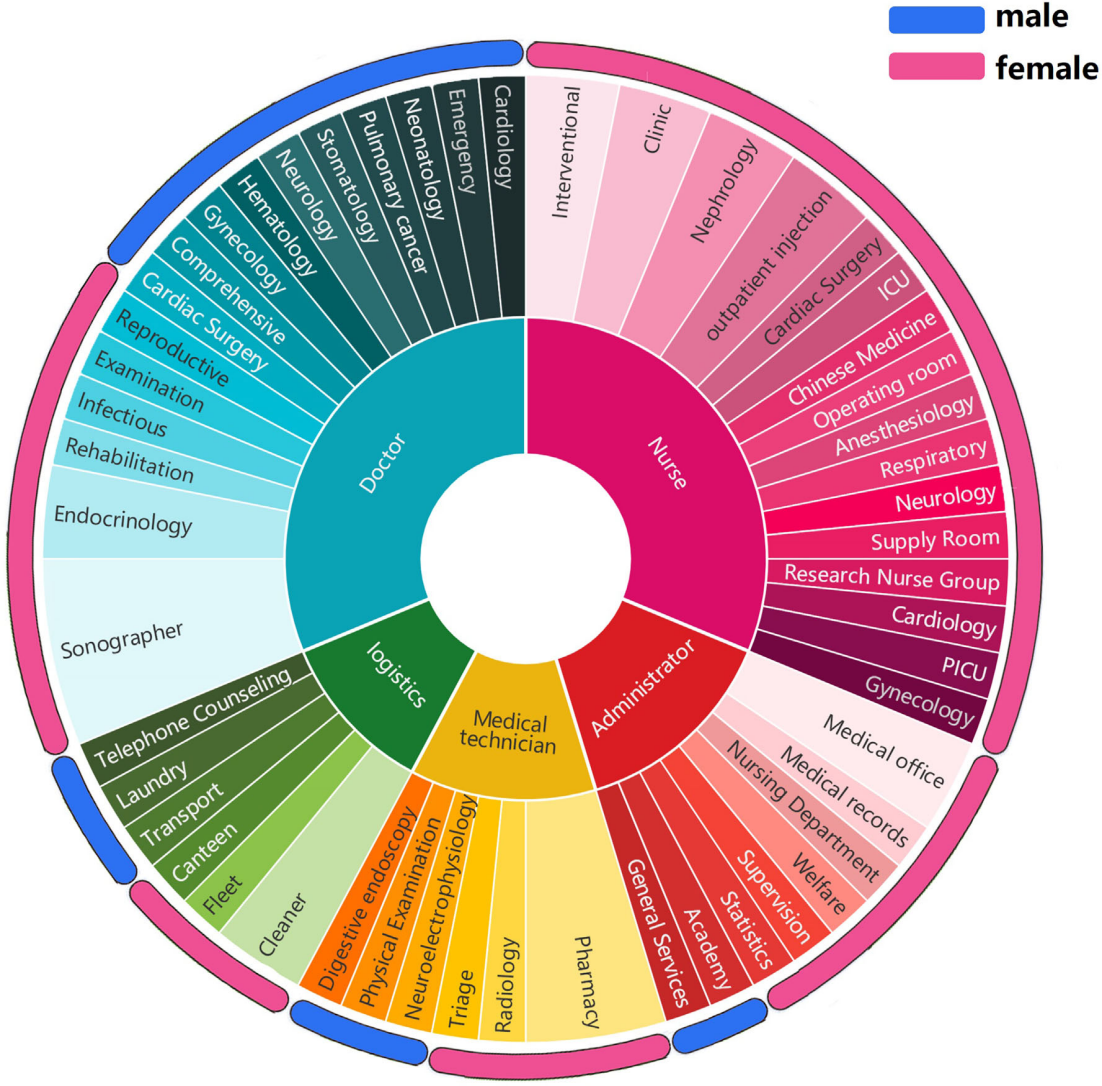
Distribution of occupational types/departments and the ratio of male to female of lung cancer incidence in the hospital population.

As of 2021, according to the screening data of LDCT staff in our hospital, the number of doctors, nursing staff, medical technicians, administrative staff, and logistics staff ages ≥40 was 874, 893, 307, 167, and 311, respectively, of which doctors diagnosed 34 people with lung cancer (incidence rate, 3.89%), 35 people were diagnosed with lung cancer by nursing staff (incidence rate, 3.92%), 13 people were diagnosed with lung cancer by medical technicians (incidence rate, 4.23%), and 8 people were diagnosed with lung cancer by administrative staff (incidence rate, 4.79%). Thirteen logisticians were diagnosed with lung cancer (incidence rate, 4.18%), and there was no significant difference (*p* = 0.184) Among them, 63 (4.1%) of 1523 female employees were diagnosed with lung adenocarcinoma, and 25 (2.4%) of 1029 male employees were diagnosed with lung adenocarcinoma. There was a significant difference in the incidence of lung cancer between men and women (*p* = 0.002) (Figure 7).

### Risk factors of lung cancer group and under following group compared with healthy people

A total of 521 people participated in the questionnaire survey, 466 valid questionnaires, of which 22 were filled out by pathologically diagnosed lung cancer patients, 124 people were followed up with nodules, and 320 people had no suspicious lung nodules detected (Table 4).

**TABLE 4:** χ^2^ analysis of risk factors for lung cancer and suspicious nodules

	*n*	Nodules incidence	*p*	Lung cancer incidence	*p*
Obesity					
Yes	49	17	0.12	1	0.304
No	417	107		21	
Chronic cough					
Yes	13	4	0.47	1	0.417
No	453	120		21	
Hemoptysis					
Yes	3	0	0.394	0	0.865
No	463	124		22	
COPD					
Yes	10	6	0.25	4	0.101
No	456	118		18	
Long‐term hormones					
Yes	9	3	0.445	1	0.355
No	457	121		21	
Family history					
Yes	129	33	0.426	4	0.223
No	337	91		18	
Smoking					
Yes	35	10	0.46	1	0.496
No	431	114		21	
Secondhand					
Yes	125	30	0.258	5	0.435
Smoking					
No	341	94		17	
Surgical smoke					
Yes	40	15	0.077	2	0.58
No	426	109		20	
Ionizing					
Yes	77	23	0.282	3	0.493
Radiation					
No	389	101		19	
Radon					
Yes	16	4	0.572	0	0.455
No	450	120		22	
Asbestos					
Yes	7	1	0.404	0	0.711
No	459	123		22	
Inorganic Kr					
Yes	9	2	0.555	0	0.645
No	457	122		22	
Tar					
Yes	45	10	0.306	2	0.641
No	421	114		20	
Chloromethyl					
Yes	12	3	0.599	0	0.556
No	454	121		22	
Long‐term					
Yes	327	83	0.21	16	0.5
Cooking					
No	139	41		6	
Junk food					
Yes	104	18	0.009	1	0.026
No	362	106		21	
Vegetable					
Yes	451	120	0.598	22	0.479
No	15	4		0	
Dairy					
Yes	368	98	0.548	16	0.307
No	98	26		6	
Formaldehyde					
Yes	189	53	0.318	9	0.569
No	277	71		13	
Trauma					
Yes	81	22	0.5	3	0.449
No	385	102		19	
High pressure					
Yes	200	53	0.525	6	0.095
No	266	71		16	

### Distribution of PM2.5 in the hospital

**FIGURE 8:**
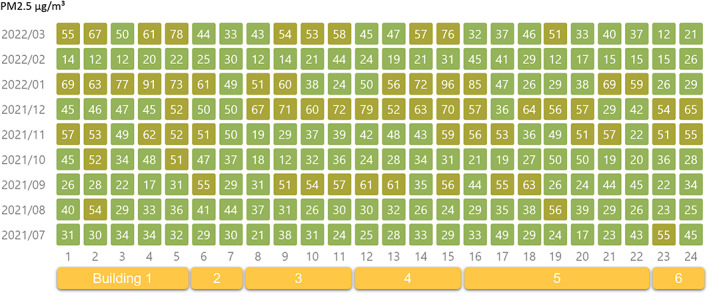
Monthly mean value of PM2.5 at each monitoring point in different buildings.

**FIGURE 9:**
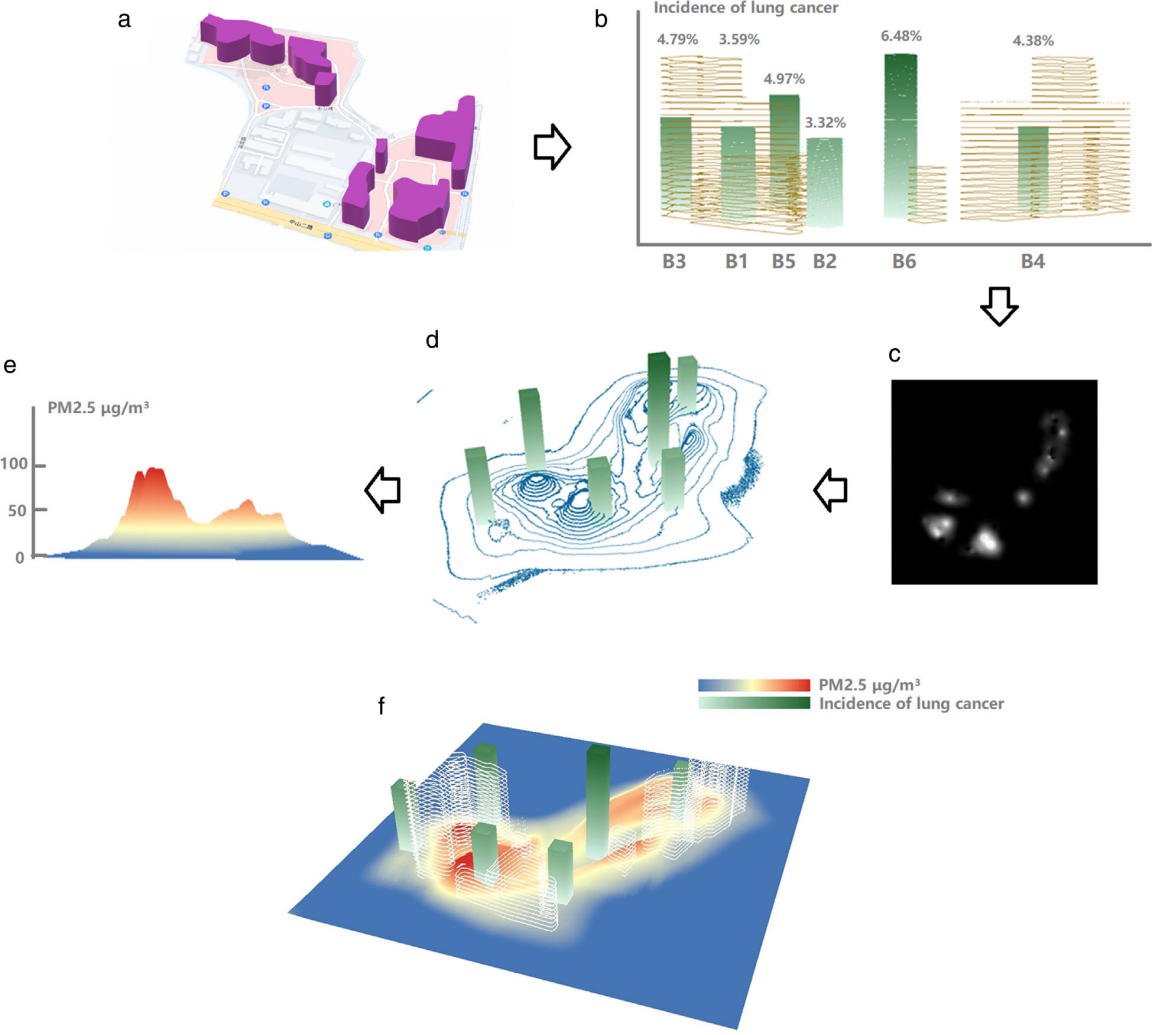
Dust concentration map with the building location and the incidence of lung cancer. (a) Adjacent relationship and topographic map of hospital building distribution, (b) histogram of lung cancer incidence between different buildings, (c) grayscale map generated by dust monitor based on PM2.5 concentration, (d) 25 monitors through equal the gray points are connected to form a PM2.5 concentration contour map. (e) Generate a plane heat map based on the contour map visualization. (f) Combine the topographic map to form a visual schematic diagram of the fitting of the three‐dimensional dust concentration map with the building location and the incidence of lung cancer.

**FIGURE 10:**
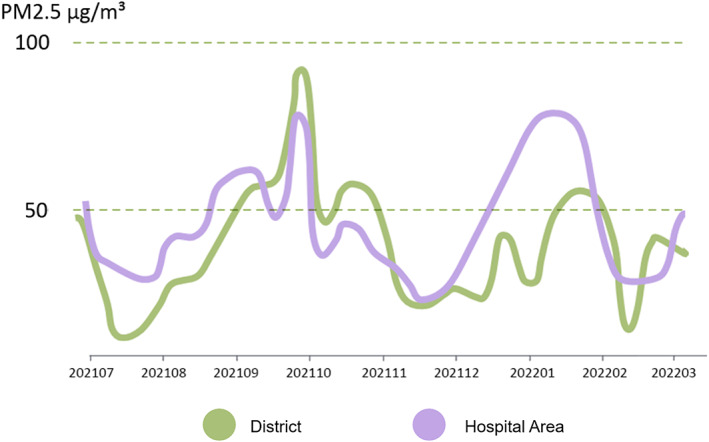
Line chart of PM2.5 in the hospital area compared to the district where the hospital is located.

No significant difference was found between office buildings and the daily average PM2.5 concentration (Table 5, Figures 8, 9, 10).

**TABLE 5:** The relationship between lung cancer diagnosis rate and PM2.5 in different areas

	Daily average PM2.5 μg/m^3^	Lung cancer incidence
Budling 1	62	3.59% (42/1169)
Budling 2	24	3.32% (16/482)
Budling 3	58	4.79% (16/334)
Budling 4	96	4.38% (6/137)
Budling 5	43	4.97% (16/322)
Budling 6	38	6.48% (7/108)

*Note*: Budling 1‐5 are independent building in the hospital.

### Ionizing radiation exposure values of different radiation‐related jobs

For 30 volunteers engaged in ionizing radiation related medical were asked to long‐term carry a personal radioactivity measurement material (Thermo Scientific TLD) for dynamic monitoring of radiation exposure during work, and it would sound an alarm when the radiation exceeds a safe value. The total annual dose intake is calculated by multiplying the daily working hours intake by the number of working days per year.

**FIGURE 11:**
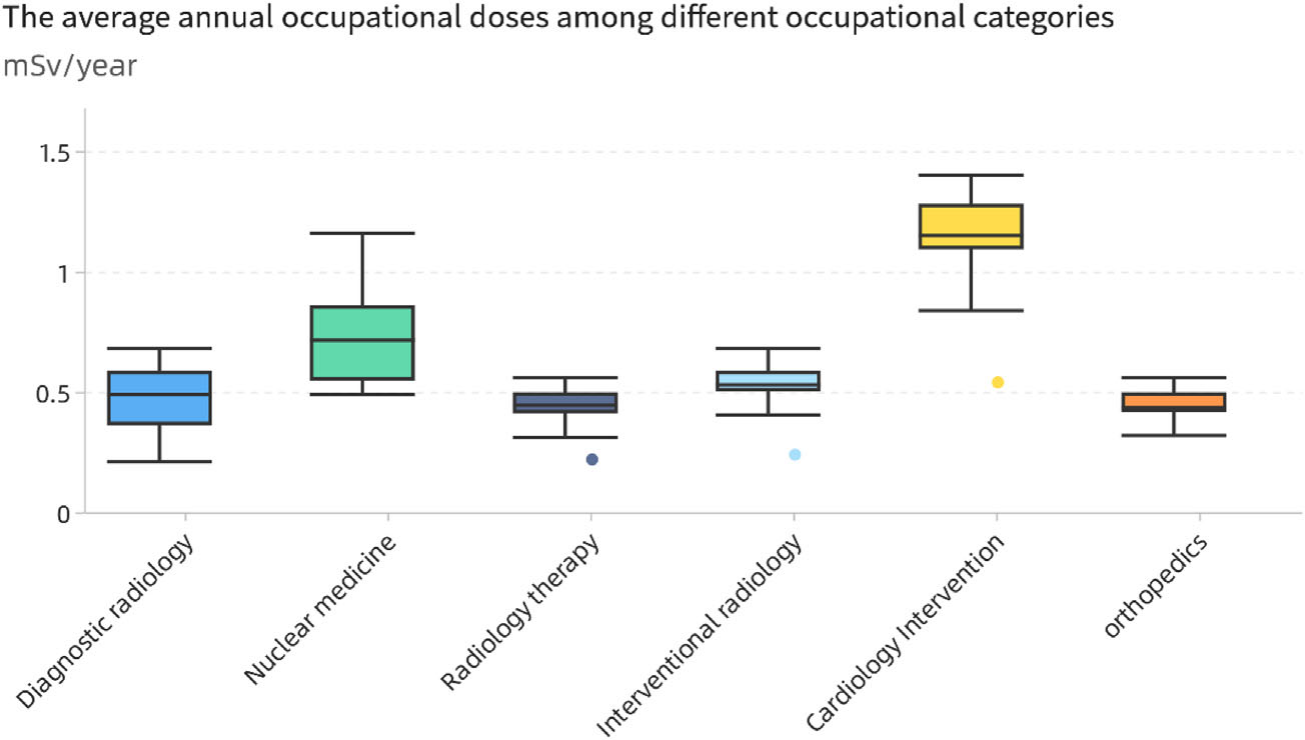
Quantitative box plot of annual ionizing radiation uptake for different radiation‐related occupations.

Among them, 10 were engaged in radiological diagnosis (without exposure to ionizing radiation equipment, as a negative control), seven were in radiotherapy, four were in interventional therapy, four were in radioisotope application, and five were in orthopedics. The medical staff in the radiation group wore special equipment in accordance with national regulations to avoid damage caused by ionizing radiation. The average annual occupational doses among radiological diagnosis, nuclear medicine, radiology therapy, interventional radiology, cardiology intervention, and orthopedics are 0.50, 0.74, 0.48, 0.54, 1.23, 0.47 mSv/year, respectively (Figure 11). All volunteers were within the legal safety ionizing radiation threshold range, none of them detected a suspicious nodule and the incidence of lung cancer among radiation‐related colleague is not found to be higher than that of non‐radiation‐related colleague.

## DISCUSSION

### The popularity of screening has led to an increase in the incidence of lung cancer, but it does not mean that it is excessive

In recent years, the number of patients with multiple pulmonary nodules has increased, and there are still many difficulties in the clinical diagnosis and treatment of such patients. It is difficult to balance the potential long‐term survival benefit and loss of lung function after nodule resection, especially for pure ground glass nodules, which have specific imaging and pathological basis. Alveoli have nice light transmittance regardless of whether it is filled with cellular components or liquid components and they will show ground‐glass nodules on CT scans. Therefore, the imaging and pathological basis of ground‐glass nodules are different from solid nodules. It can also be a precancerous lesion of cell proliferation, or even an early manifestation of a rapidly progressing adenocarcinoma.

According to the NLST trial data, despite the high detection rate of pulmonary nodules, only a small proportion (~4%) of pulmonary nodules are now found to be malignant lesions through large‐scale screening. The question is how to accurately identify malignant nodules in a large number of patients with pulmonary nodules found clinically? This is also an important problem faced by doctors in the stage of clinical treatment.

The reason for the increase in the incidence of lung cancer is because of the advancement of imaging equipment and the artificial intelligent (AI) nodule recognition and outline, but at the same time, it has also brought many false positive results, increased the psychological burden of the public, and also induced radiologists or surgeons to over diagnosis and overtreatment. The popularization of screening and the development of imaging equipment have led to an increase in the detection rate of lung cancer, but the current prevalence is closer to the true number of lung cancer in the population. For indolent lung cancer (AIS/MIA), long‐term survival with tumor may be possible, but rapidly progressive lung cancer also has ground‐glass morphology in the early stage, and ground‐glass nodules found during screening do not mean that they are definitely indolent nodules. It is very important to distinguish active nodules that require early intervention from indolent GGOs that can be followed up for a long time, which can greatly save medical resources, social medical insurance, and reduce unnecessary surgical treatment.

### Correlation between air quality and lung cancer incidence

As far as the current situation is concerned, a large number of epidemiological studies have suggested that various particulate matters contained in air pollution are inextricably linked to lung cancer. On the basis of the original research, 18 new studies around the world have analyzed the correlation between PM2.5 and lung cancer. The final results showed that for every 10 μg/m^3^ increase in fine particulate matter, the probability of an individual suffering from lung cancer increased by 9%. According to the latest research results released by the WHO, outdoor air pollution is the primary environmental factor leading to the death of patients with lung cancer, which must be given full attention. In 2014, the International Agency for Research on Cancer (IARC) officially listed outdoor air pollution as a first‐class carcinogen. Air pollutants mainly include fine particles (PM2.5) and inhalable particles. PM2.5 may produce corresponding toxins at different levels of genetic material, resulting in obvious changes in the internal chromosome structure of the human body, and eventually lead to gene mutation and DNA damage and other corresponding diseases. PM2.5 is as harmful to Chinese residents as smoking. However, because of the different susceptibility of the population, non–small cell lung cancer, especially adenocarcinoma, is more common in women and non‐smokers in China. Studies by related scholars have shown that the incidence of lung adenocarcinoma is inextricably linked with the air pollution index.

The PM2.5 monitoring reflects the situation of the atmosphere over time. In fact, lung cancer is the result of a long‐term interaction with environmental pollution that may not emerge until decades later. Therefore, the PM2.5 should be monitored continuously, and the correlation with lung cancer will be analyzed from the contemporary staff many years later.

### Correlation between radiation pollution and lung cancer incidence

According to the recommendations of the International Commission on Radiological Protection (ICRP) and China's basic standards for radiation health protection (GB‐4792‐84) employees exposed to radioactive machines should not exceed 20 mSv/year (10 μSv/h), and general individuals should not exceed 1 mSv/year (0.52 μSv/h).

Ionizing radiation, such as gamma rays, X‐rays, and radioactive particles, can cause cancer by damaging DNA. However, how tumors are related to radiation damage to genetic material remains unclear. All personnel currently participating in screening are within legal thresholds. Occupational radiation exposure control in the hospital is well‐controlled, and staff are working in a relatively safe environment. Accurate, radiation monitoring materials are not carried around the body for 24 hours. We found no difference in lung cancer incidence between radiation‐related and non‐radiation‐related workers.

Because of the complex environment in the hospital, risk factors are hard to discover and monitor, and the questionnaire survey involving personal privacy and valid questionnaires is too small to reflect real data. Eventually, no exact lung cancer risk factors have been found, indirect effects or confounded effects of multiple factors, and larger epidemiological investigations are needed to identify potential risks.

### Adjust the definition of risk population and control over diagnosis and overtreatment

In 103 patients with lung adenocarcinoma, only one high risk individual according to NCCN recommendations. The so‐called early screening is the screening of patients without lung cancer‐related symptoms, but with risk factors. However, there are still more than 20% of lung cancer patients without a history of smoking. Moreover, they may be in an environment of passive smoking for a long time, and the impact of second‐hand smoke on health is no less than that of active smokers; moreover, traditional cooking methods such as frying and deep‐frying produce kitchen fumes that contain more than 200 harmful substances, including a variety of carcinogens. People who cook for a long time should also belong to the high‐risk group of lung cancer. In clinical, we can clearly realize that the incidence of lung cancer in non‐smokers is increasing. Chen Haiquan and his team^43^ have held a large‐scale LDCT early screening, among employees in Shanghai communities and six hospitals in different regions; they have successively revealed Chinese female non‐smokers have a higher incidence of lung cancer. For low‐risk groups, low frequency lung cancer screening should be recommended. From the results of current epidemiological studies, it is necessary to conduct in‐depth research on the risk factors of lung cancer in non‐smoking populations, and conduct large‐scale prospective clinical trials to evaluate non‐smoking populations for lung cancer. However, it should be noted that risk factors such as second‐hand smoke, indoor and outdoor air pollution, and occupational exposure related to non‐smokers are very difficult to quantify. In addition, early‐stage cancer screening programs often require long follow‐up times to assess benefits and risks, and we may not be able to determine the benefits of early‐stage lung cancer screening in this population in a short period of time.

In conclusion, the high screening rate brings about a large number of lung cancers being detected. Increasing number of lung cancer has been found in non‐high‐risk groups, current screening strategies need to be adjusted. Further research on the pathogenesis of lung cancer in female non‐smokers is crucial. An efficient pathological prediction model to distinguish active nodules from GGOs is required.

## CONFLICT OF INTEREST

No potential conflict of interest was reported by the authors.
